# Inferior mesenteric vein preserving lymphadenectomy in high left segmental colectomy for splenic flexure melanoma: A case report

**DOI:** 10.1016/j.ijscr.2023.107956

**Published:** 2023-03-02

**Authors:** Amy Crowe, Ra Nasser, Ishith Seth, Angus Lee

**Affiliations:** a Department of General Surgery, Bendigo Health, Victoria 3550, Australia; b Department of Surgery, The University of Melbourne Medical School, Victoria 3052, Australia; c Central Clinical School, Monash University, Victoria 3004, Australia

**Keywords:** Splenic flexure, Cancer, Melanoma, Colectomy, Inferior mesenteric vein

## Abstract

**Introduction and importance:**

Surgical resection is the mainstay for management of splenic flexure cancers, with the aim of achieving adequate lymphadenectomy. Left-sided bowel resections often require ligation of the inferior mesenteric vein (IMV) for mesocolic dissection or lymphadenectomy which can result in congestive colitis on the anal side of the anastomosis secondary to poor venous outflow. Preserving the IMV may mitigate this risk but is technically difficult and can compromise oncological resection. This case report is a rare example of high left segmental resection of the splenic flexure with preservation of the IMV in a patient with splenic flexure melanoma.

**Case presentation:**

A non-obstructing lesion was discovered in a 73-year-old male who underwent colonoscopy following a positive faecal occult blood test. Biopsy of the lesion confirmed a melanoma. This patient had a history of cutaneous melanoma which was excised 20 years prior. A laparoscopic high left segmental colectomy was performed, and metastatic melanoma was identified in 3 of 12 regional lymph nodes. The patient recovered with no complications.

**Clinical discussion:**

This patient underwent high left segmental colectomy to achieve oncological clearance while resecting minimal bowel and preserving bowel function. The IMV was spared in this surgery to prevent venous congestion. Reports of colitis following left sided colectomy have been described, whereby colitis is thought to result from a mismatch in arterial perfusion and venous drainage following IMV resection.

**Conclusion:**

This case highlights the potential role of preservation of the inferior mesenteric vein in a rare case of splenic flexure melanoma.

## Highlights

• Preservation of inferior mesenteric vein in left-sided colectomy is rare and adequate lymph node retrieval is important.
• Preservation of inferior mesenteric vein in left-sided colectomy allows to prevent congestion and colitis.
• Multi-disciplinary discussion for splenic flexure carcinomas are indicated.

## 1 Introduction

Splenic flexure carcinomas (SFCs) represent 2–8 % of all colorectal tumors. Although there is literature available on the management of SFCs, there is no worldwide consensus on optimal surgical management [1]. Currently, surgical treatment includes extended right hemicolectomy, left hemicolectomy, subtotal colectomy, or segmental colectomy [1], [2], and considers the anatomy, vascular supply and lymph node drainage of mid-gut and hind-gut structures. Retrospective studies suggest no differences in survival outcomes or post-operative complications between surgical approaches, therefore treatment is case-based [2], [3], [4], [5], [6].

Left-sided colectomy typically resects the inferior mesenteric vein (IMV) which reduces portal venous drainage of the colon, potentially resulting in venous congestion. Multiple reports demonstrate delayed onset regional congestive colitis on the anal side of the anastomosis after left-sided colon cancer surgery, where IMV ligation is postulated to disrupt colonic microcirculation [7], [8], [9]. Adequate lymphadenectomy during mesocolic resection with preservation of the IMV has been reported in two cases however the difficulty with this approach is to ensure adequate lymph node retrieval for oncological resection alongside a technically challenging dissection [10], [11]. This case report provides an example of adequate lymph node dissection which preserved the IMV during a high left segmental colectomy in a patient who presented with isolated melanoma of the splenic flexure. Appropriate written and verbal consent was obtained from the patient and the case report followed the SCARE guidelines [12].

## 2 Presentation of case

**Fig. 1:**
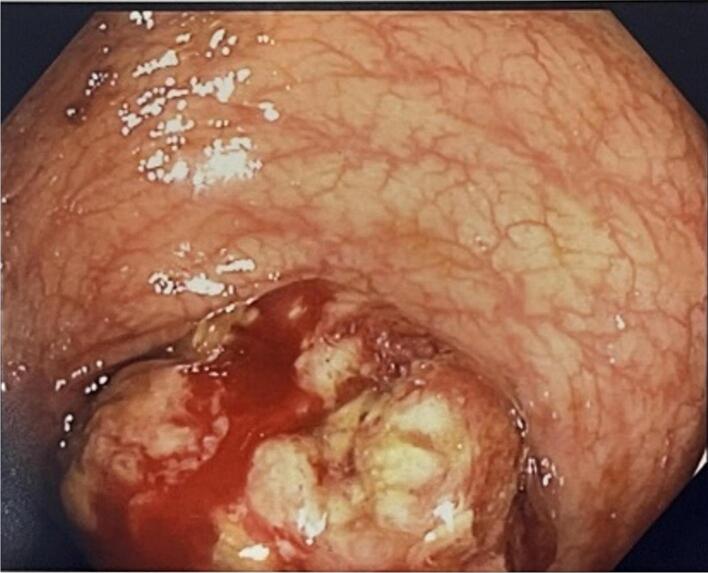
Mucosal lesion identified with colonoscopy.

**Fig. 2:**
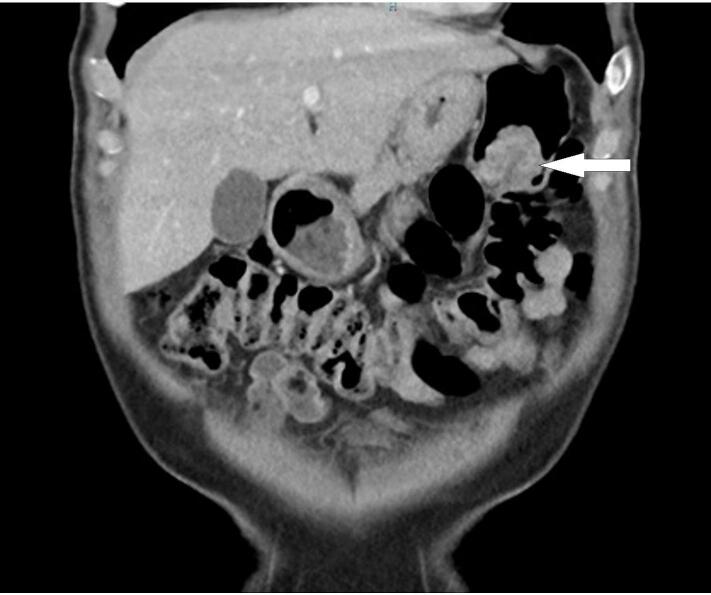
Coronal CT image identifying lesion in the splenic flexure (white arrow).

**Fig. 3:**
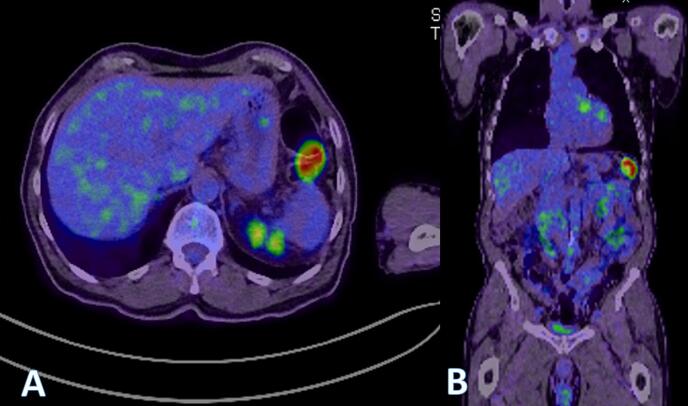
A) Axial PET scan identifying avid lesion in the splenic flexure, B) coronal section of PET scan identifying the lesion in the splenic flexure.

A 73 year old male underwent a colonoscopy for a positive faecal occult blood test. His past medical history includes melanoma (Clark level 4) of the back which was completely excised 20 years prior, and hypertension. A non-obstructing lesion was found at the splenic flexure (Fig. 1) and tattooed. Biopsy at colonoscopy showed melanoma and the patient completed staging with MRI brain, PET CT and CT chest, abdomen and pelvis which identified the isolated lesion in the splenic flexure with no evidence of other metastatic diseases (Fig. 2, Fig. 3). The patient proceeded to undergo a laparoscopic high left segmental colectomy with preservation of the IMV. Histopathology of the operative specimen, a segment of colon 200 mm in length, confirmed a 30 mm melanoma penetrating through the bowel wall with clear margins. Metastatic melanoma was identified in 3 of the 12 retrieved regional lymph nodes. B-RAF mutation was detected through immunohistochemistry.

Following surgical resection, the patient recovered without complications was content with the management and was discharged home four days post operatively after regaining bowel function. Following a discussion at a multi-disciplinary meeting, the patient opted for surveillance as an alternative to adjuvant immunotherapy based on the potential risk profile of the treatment and unknown benefits. The initial plan for surveillance included a clinical review and 3 monthly PET and MRI scan, however the patient was unable to maintain ongoing imaging after 6 months due to the high burden of appointments and travel time required. Colonoscopy at 12 months post-operatively showed a single benign polyp. At 18 months post-operatively no signs of recurrence were observed, and clinical surveillance is ongoing.

### 2.1 Technical component

**Fig. 4:**
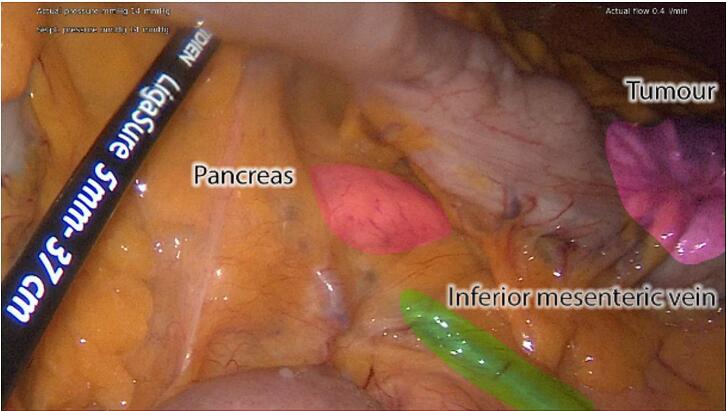
Intra-operative image demonstrating key anatomy, the inferior mesenteric vein highlighted to show its path superiorly towards pancreas.

The procedure took place in a modified Lloyd-Davis position with the right arm tucked in. Infraumbilical 10 mm Hasson port was placed and 14 mmHg pneumoperitoneum was established. Three 5 mm working ports were inserted in the RUQ, LUQ, and LLQ. Fig. 4 identifies key anatomical structures where the IMV drains towards the inferior border of the pancreas.

Following the identification of the IMV we utilised a sub-IMV approach with medial to lateral dissection to enter the plane between the mesocolon and Gerotas fascia. This was performed using a combination of blunt dissection and Ligasure. The gonadal vessels lie deep to the plane of dissection. The ascending branch of the left colic artery was ligated as skeletonization of the IMV continued towards the inferior border of the pancreas.

**Fig. 5:**
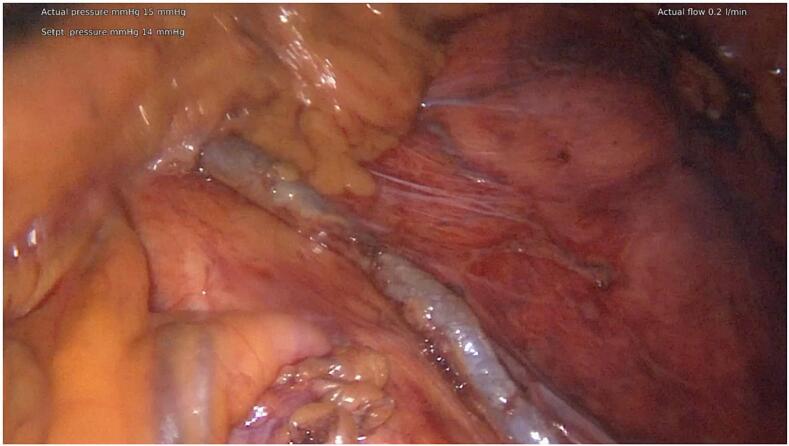
Intra-operative image of skeletonised inferior mesenteric vein following mesocolic dissection.

**Fig. 6:**
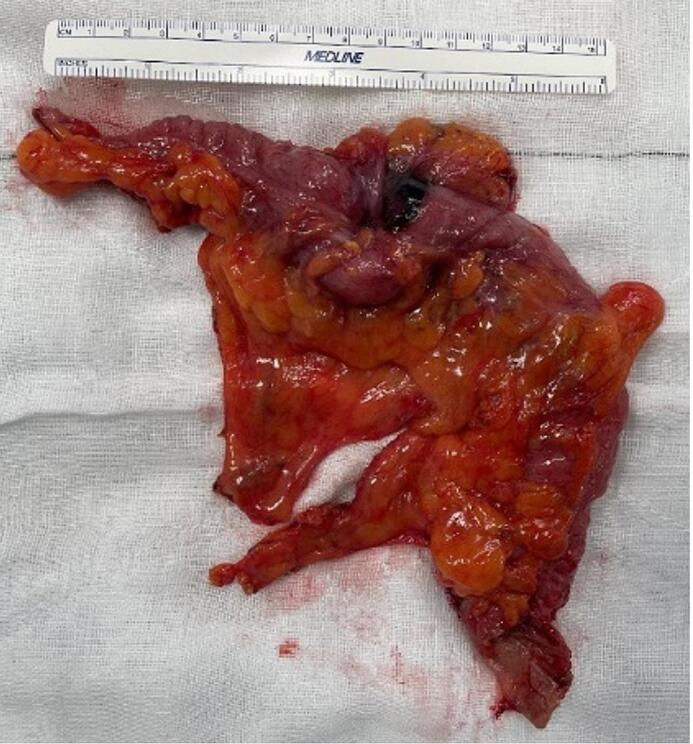
The operative specimen following segmental colectomy.

A window into the lesser sac was created with an infra-colic approach through the transverse mesocolon to the right of middle colic artery. The left branch of the middle colic artery was ligated with Ligasure while the remaining colon and sigmoid colon were further mobilised medially to allow for tension-free extra-corporeal anastomosis (side to side stapled). Fig. 5 shows a skeletonised IMV. The specimen (Fig. 6) was extracted through a small Alexis wound retractor. The operative procedure can be seen in the supplementary video file.

## 3 Discussion

The splenic flexure is situated at a watershed area between the mid-gut and hindgut. The splenic flexure receives vascular tributaries from the superior mesenteric artery (SMA) and inferior mesenteric artery (IMA), although Griffiths et al. discovered the splenic flexure received most of its blood supply by tributaries of the IMA, with just 11 % supplied by the left branch of the middle colic artery, a branch of SMA [5], [13]. Lymph node drainage from the splenic flexure is variable and its pattern is uncertain, although malignancy is thought to spread predominantly along the paracolic arcade, left branch of the middle colic artery, left colic artery, and towards the root of the IMV [2].

**Fig. 7:**
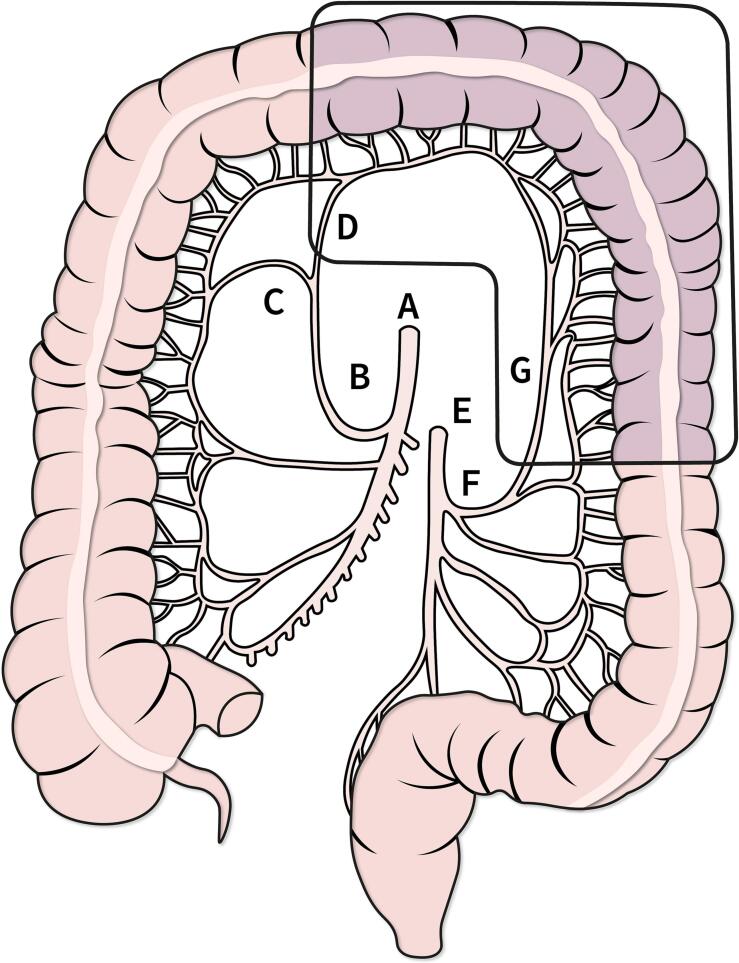
Schematic of arterial supply to the colon and resection specimen. A. Superior mesenteric artery B. Middle colic artery C. Right branch of the middle colic artery D. Left branch of the middle colic artery E. Inferior mesenteric artery F. Left colic artery G. Ascending branch of the left colic artery.

Surgical resection must incorporate resection of the malignancy with a margin of healthy tissue, in addition to that necessitated by arterial ligation and adequate lymphadenectomy. A recent meta-analysis of non-randomised studies indicated no significant difference in short term surgical complications or oncological outcomes between common surgical resection options [2], leaving no clear pathway for surgical resection of splenic flexure cancers [1], [4], [5], [6]. For this patient, a high left segmental colectomy was performed, requiring ligation of the left branch of the middle colic artery and the ascending branch of the left colic artery (Fig. 7).

Multiple reports of colitis following left sided colectomy have been published [7], [8], [9]. These reports postulate that congestive colitis may occur when there is a mismatch in arterial perfusion and venous drainage, however the number of cases is low and therefore the full scope of the impacts of vessel ligation or preservation on patient outcomes is not well understood. Which vessels to ligate will depend on the area of resection and the ability to achieve adequate lymphadenectomy while avoiding surgical complications secondary to reduced perfusion such as ischemic colitis or anastomotic leakage. Fujii et al. describe four cases of delayed congestive colitis at the anal side of anastomosis out of 191 patients who underwent left hemicolectomy whereby the superior rectal artery was preserved and the IMV was dissected at the inferior margin of the pancreas [7]. Inoue et al. also reported a case of chronic progressive colitis following sigmoidectomy with sparing of the superior rectal artery and ligation of the IMV [8]. Therefore, preservation of the IMV in procedures that preserve the SMA and IMA may help prevent venous congestion, however dissection around the IMV during lymph node retrieval is technically difficult [7]. This case report demonstrates the feasibility of IMV preservation without compromising oncological principles, however further prospective studies would aid in validating the feasibility and safety of this technique on a larger scale.

The patient in this case has a history of cutaneous melanoma Clarke level IV. Histopathology of the operative specimen indicated a 30 mm ulcerated melanoma (SOX-10 positive, BRAF positive) penetrating through bowel wall and present in 3/12 lymph nodes. It is not known whether this lesion represents a primary mucosal melanoma or a recurrence of his previous cutaneous melanoma. Regardless, operative intervention is recommended for metastatic melanoma to the GI tract for both symptom management and to prolong survival [14], [15]. Metastatic cutaneous melanoma in the colon is rare and is usually diagnosed on autopsy [16], [17]. Primary mucosal melanomas are rare (1.4 % of all melanomas), and mucosal melanomas of the colon are rarer still – accounting for 0.9 % of all mucosal melanomas [18]. Positive B-RAF mutation is present in 4 % of cases of mucosal melanoma in the lower GI region (lower GI tract, genital, anorectal) [19]. This may support the diagnosis of metastatic cutaneous melanoma. Whilst metastasis to the bowel 5 years following initial treatment has been reported [16], [20], to the authors knowledge this would be the first report of metastasis to the colon found pre-mortem 20 years following initial management.

## 4 Conclusions

This case report demonstrates a rare case of melanoma of indeterminate origin in the colon, and considers the technical difficulty and potential role of IMV preservation in colectomy. Furthermore, this case report highlights the paucity of literature on the surgical management of splenic flexure carcinomas and emphasizes that worldwide consensus is needed for optimal management.

## Abbreviations

IMV: inferior mesenteric vein
IMA: inferior mesenteric artery
SMA: superior mesenteric artery

The following are the supplementary data related to this article.

Supplementary Video 1 Colonic melanoma. Supplementary Video 1

## Patient consent

Written informed consent was obtained from the patient for publication of this case report and accompanying images. A copy of the written consent is available for review by the Editor-in-Chief of this journal on request.

## Sources of funding

None.

## Ethical approval

The ethics was exempt from ethical approved due to being a case report, aligned with institutional values (https://www.bendigohealth.org.au/Ethicsapplications/).

## Author contribution

Amy Crowe: Study concept and design, literature review, manuscript writing, manuscript review and editing.

Ra Nassar: Manuscript review and editing, pictorial editing.

Ishith Seth: Manuscript review and editing.

Angus Lee: Supervising surgeon, study concept and design, manuscript review and editing.

## Guarantor

Dr. Ishith Seth, BBiomed(Hons), MD.

## Research registration

N/A.

## Declaration of competing interest

None declared.
